# Exosomes Derived From Mesenchymal Stromal Cells Pretreated With Advanced Glycation End Product-Bovine Serum Albumin Inhibit Calcification of Vascular Smooth Muscle Cells

**DOI:** 10.3389/fendo.2018.00524

**Published:** 2018-09-21

**Authors:** Ying Wang, Wen-Qi Ma, Yi Zhu, Xi-Qiong Han, Naifeng Liu

**Affiliations:** Zhongda Hospital, Southeast University, Nanjing, China

**Keywords:** advanced glycation end products, vascular calcification, BMSC, exosome, miR-146a, TXNIP

## Abstract

**Background:** The osteogenic differentiation of vascular smooth muscle cell (VSMCs) is important for the development of vascular calcification (VC), particularly in diabetes. Exosomes derived from Mesenchymal Stromal Cells (MSCs) are effective against cardiovascular diseases, yet their role in VC remains unclear. Advanced glycation end products (AGEs) inhibit bone marrow stromal cell osteogenesis by targeting osteogenesis-associated genes. Thus, we investigated the role of exosomes derived from MSCs pretreated with AGEs-BSA in VC and its potential mechanisms.

**Methods:** Primary VSMCs and MSCs were isolated from the aorta and bone marrow of Sprague-Dawley rats, respectively. VSMCs were cultured with AGEs-BSA to induce osteogenic differentiation. Exosomes were harvested from MSCs by ultracentrifugation. MSCs and VSMCs were cocultured in Transwells, and exosomes were added to VSMC culture medium to assess their effects on osteogenic differentiation. Double luciferase reporter assay was applied to confirm that miR-146a directly targets the 3' UTR of the thioredoxin-interacting protein (TXNIP) gene.

**Results:** Pretreatment of VSMCs with AGEs-BSA increased the expression of thioredoxin-interacting protein (TXNIP) by inhibiting that of miR-146a, resulting in enhanced ROS production and VSMC calcification. By contrast, the expression of miR-146a in MSCs was increased by AGEs-BSA treatment. Thus, miR-146a was transferred from AGEs-BSA-pretreated or miR-146a-transfected MSCs to VSMCs via exosomes. After coculture with miR-146a-containing exosomes, the AGEs-BSA-mediated increase in VSMC calcification was diminished, accompanied by decreased TXNIP expression and ROS production. Furthermore, TXNIP overexpression counteracted the anti-calcification effects of MSC-derived miR-146a-containing exosomes. In addition, TXNIP was identified as a target gene of miR-146a, and the results of double luciferase reporter assay confirmed that TXNIP was the direct target gene of miR-146a.

**Conclusions:** Exosomes secreted by MSCs pretreated with AGEs-BSA contained a high level of miR-146a, which was transferred to VSMCs and inhibited AGEs-BSA-induced calcification in a TXNIP-dependent manner. Thus, miR-146a-containing exosomes may be a potential therapeutic target for VC.

## Introduction

Vascular calcification (VC), which is characterized by the deposition of calcium phosphate in cardiovascular structures, is associated with an increased risk of cardiovascular events and mortality (1). VC is highly prevalent in patients with diabetes, but its mechanism is multifactorial and incompletely understood (2, 3). Phenotypic transition of vascular smooth muscle cells (VSMCs) into osteoblast-like cells, which is characterized by upregulation of osteogenic-related gene expression, is now seemed as a critical pathological process in vascular calcification (4).

Advanced glycation end products (AGEs), which are generated by a non-enzymatic reaction between proteins and sugar residues, are the main cause of diabetes-related vascular complications, including diabetic VSMC calcification (5). Our previous study has demonstrated that AGEs induce VSMC calcification through ROS production (6). Unfortunately, little is known about the detailed mechanism underlying, and the potential therapeutic measures for VC.

Bone marrow-derived mesenchymal stem cells (MSCs) are multipotential stem cells and have the potential to differentiate into chondrocytes and osteoblasts, and participate in the process of bone formation (7). Despite the effect of AGEs on inducing VSMC osteogenesis transformation, it played an opposite role in bone-related cells. Previous studies have revealed that AGEs are a an important promotive factor in osteoporosis (8). Thus, it is meaningful to illuminate the mechanisms underlying those contradictory phenomena.

In the field of stem-cell therapy, MSCs are considered as one of the most promising stem cell types for treating cardiovascular disease (9). The therapeutic effect of MSCs has been attributed to directional differentiation into target cell types (10), angiogenesis (11), and repairing injured tissues (12). While recent studies have considered that some of these reparative effects are mediated by paracrine factors secreted by MSCs, supported by evidences such as the effects of MSCs are mimicked by MSC conditioned medium (CM) (13).

Exosomes are nano-sized (50–200 nm) vesicles with double membranes that are secreted into the extracellular environment (14). Exosomes released from MSCs function as paracrine mediators and are responsible for the therapeutic effects of BMSCs (15). They mediate intercellular communication by transferring functional proteins, lipids, and nucleotides (16, 17). Exosomes can restore injured cardiac tissue by modulating the local microenvironment, promoting local microvascular network formation and regeneration, and regulating cell proliferation and apoptosis (18, 19). Furthermore, MSCs exosomes have been applied on many vascular disease therapies, such as cardiac fibrosis (20), cerebral infarction (21), liver ischemia/reperfusion injury (22), etc. However, to the best of our knowledge, no study has been reported regarding their role on vascular calcification.

MicroRNAs (miRNAs) are a kind of small non-coding RNAs, post-transcriptionally regulate gene expression, function by targeting the 3′-untranslated regions (3′-UTR) of mRNAs for degradation or preventing translation. Recently, evidences suggests that microRNAs can be transferred between cells through exosomes and mediate target gene repression in recipient cells (23, 24). Noteworthly, MSCs can secrete exosomal miRNAs, which was considered as an important mechanism for intercellular communication. Recent studies indicate that the loading of miRNAs into exosomes is a selective process. What's more, exogenous intervention such as drug administration or gene transfection performed on donor cells can significantly change the miRNA profile of the derived exosomes.

Increasing studies suggested that miR-146a exerted anti-oxidative stress effect in various pathological processes in diabetic complications (25–27). Wang et al. reported that miR-146a decreases high glucose induced ROS generation in endothelial cells (28). It was also demonstrated that the expression of miR-146a was down-regulated in tissues of diabetic rats (29). Furthermore, its overexpression of has protective effects in diabetic mellitus (20, 30, 31), suggest that miR-146a may serve as an important protector in diabetic condition. Despite the positive effect of miR-146a under diabetic condition, however, some studies suggested that miR-146a has a negative effect on MSC osteogenic differentiation (32). Interestingly, both AGEs-BSA and miR-146a play negative effects on MSC osteogenesis. What's more, AGEs-BSA has been reported to induce the miR-146a expression in microphage cells. Whether AGEs promote or restrain osteogenic differentiation in VSMC and MSC through miRNA-146a is an interesting questions need to be addressed.

Thioredoxin-interacting protein (TXNIP) (50 kDa) is a member of the α-arrestin family and is predicted by the miRDB to be a target gene of miR-146a^1^. TXNIP binds and inactivates thioredoxin (Trx), leading to increased reactive oxygen species (ROS) production (33). Overexpression of *TXNIP* impairs the reducing activity of Trx, thereby increasing the level of oxidative stress (34). Under oxidative stress condition, excessive ROS generation causes a conformational change in TXNIP, resulting in its dissociation from Trx (35). Since the Trx/TXNIP complex plays an important role in regulating cellular redox status, we speculated that TXNIP-related oxidative stress is involved in vascular AGEs-BSA-induced calcification.

To address this knowledge gap, we investigated the role of exosomal miR-146a in AGEs-BSA-induced calcification of VSMCs. We hypothesized that AGEs-BSA-pretreated MSC-derived exosomes contained a high level of miR-146a, which inhibited VSMC calcification. To validate these hypotheses, we detected the effect of AGEs-BSA on the expression of miR-146a in MSCs exosomes and then assessed the effect of miR-146a-containing exosomes on the VSMC calcification. We observed that AGEs-BSA-pretreated MSC-derived exosomes contained a high level of miR-146a, which inhibited VSMC calcification via TXNIP/ROS signaling pathway.

## Materials and methods

### Cell culture and identification

MSCs were isolated from Sprague-Dawley rats by whole bone marrow culture (36) and expanded in Dulbecco's Modified Eagle's Medium (DMEM) containing 10% fetal bovine serum (FBS). Cultures were maintained at 37°C in a humidified atmosphere containing 5% CO_2_ for 48 h. The medium was changed every 2–3 days. Two positive markers (CD73 and CD105) and one negative marker (CD34 and CD45) were used to identify MSCs according to the recommendations of the International Society for Cellular Therapy (Supplementary Figure 1). Rat aortic VSMCs were cultured using the tissue explant method (37). The thoracic aorta was removed from normal male Sprague-Dawley rats under sterile conditions, the fatty tissue around the artery was removed, and the media were separated and minced into small blocks, which were transferred to cell culture plates and incubated in DMEM: Nutrient Mixture F-12 1:1 (DMEM/F-12 1:1), with 10% FBS, 100 IU/mL penicillin, and 100 μg/mL streptomycin at 37°C in a humidified atmosphere of 95% air and 5% CO_2_ until the cells reached confluence. The purity of VSMC cultures was confirmed by immunocytochemical localization of α-smooth-muscle actin and smooth muscle protein 22-α (Supplementary Figure 2). Primary VSMCs and BMSCs at passages 3–8 were used in subsequent experiments.

### Measurement of VSMC viability

Cell viability was determined by the Cell Counting Kit-8 (CCK-8). VSMCs were seeded into 96-well culture plates at 2 × 10^3^ cells/well and incubated in DMEM/F12 with 10% FBS at 37°C until reaching 70% confluence. Next, the cells were incubated in serum-free medium for 24 h. The serum-starved cells were exposed to AGEs-BSA for 24, 48, or 72 h, and the viability of the VSMCs was determined by the CCK-8 assay according to the manufacturer's instructions.

### Isolation and identification of exosomes

Exosomes were obtained from MSC culture supernatants by ultracentrifugation as previously described (38). MSCs were cultured for 48 h in DMEM with 10% exosome-depleted FBS (Gibco, Gaithersburg, MD, USA), and the cultures were centrifuged to harvest the supernatant. The supernatants were centrifuged at 3,000 g for 45 min, passed through a 0.22 μm filter to remove debris, and centrifuged at 200,000 g (Beckman Coulter) for 2 h at 4°C. The supernatants were discarded, and the pellets were washed in PBS, re-suspended, and centrifuged at 200,000 g for 2 h at 4°C. MSC-derived exosomes were characterized by transmission electron microscopy, Nanosight analysis and subjected to Western Blot analyses of CD9, Alix, and TSG 101 (Abcam, UK).

### PKH26 labeling and analyses

Exosomes were stained with the PKH26 Red Fluorescent Cell Linker Kit according to the manufacturer's instructions (Sigma, St. Louis, MO, USA). The labeled exosomes were incubated for 24 h in the presence of cells and viewed under a confocal microscope.

### Plasmid extraction and transfection

miR-146a and TXNIP-expression plasmids were constructed by Vigene Bioscience (Shandong, China). Plasmids were extracted using a Plasmid Miniprep Kit (Axygen, Hangzhou, China), and incubated with lipo2000 for cell transfection using a non-target control (NTC) miRNA as a control.

### Quantification of mRNA and miRNA levels

Total RNA was extracted from cells using TRIzol reagent (Life Technologies Corporation, Carlsbad, CA) according to the manufacturer's instructions and purified using the RNeasy Mini Kit (Qiagen, Hilden, Germany). miR-146a was extracted from exosome suspensions using the miRNeasy Serum/Plasma Advanced Kit (Qiagen). Extracted RNA was reversed-transcribed into cDNA with TaqMan Universal Master Mix II or TaqMan MicroRNA Reverse Transcription Kit (Thermo Fisher Scientific, Waltham, MA, USA), and quantitative polymerase chain reaction (qPCR) was performed using the ViiA7 Real-Time PCR System (Applied Biosystems, Foster City, CA, USA) and the QuantiNova SYBR-Green PCR Kit according to the manufacturer's instructions. Triplicate reactions were performed for each sample. Data were analyzed by the 2-ΔΔCt method and normalized to the expression of β-actin or U6 snRNA. The primers used are listed in Table 1.

**Table 1:** Primer sequences.

**Primer**		**Sequence**
miR-146a	Forward	5′-UGAGAACUGAAUUCCAUGGGUU-3′
	Reverse	5′-CCCAUGGAAUUCAGUUCUCAUU-3′
Runx2	Forward	5′-TTGGAATCGATGGTAATTATCTTTAG-3′
	Reverse	5′-AATCCTATTGCGGTAATCTTACCTTAAT-3′
BMP-2	Forward	5′-GGCTGTATTCCCCTCCATCG-3′
	Reverse	5′-CCAGTTGGTAACAATGCCATGT-3′
TXNIP	Forward	5′-TCATGGTGATGTTCAAGAAGATC-3′
	Reverse	5′-ACTTCACACCTCCACTATC-3′
β-actin	Forward	5′-GGCTGTATTCCCCTCCATCG-3′
	Reverse	5′-CCAGTTGGTAACAATGCCATGT-3′

### SDS-page and western blotting

Total cellular proteins were resolved in 10% sodium dodecyl sulfate-polyacrylamide gel electrophoresis (SDS-PAGE) gels and transferred to polyvinylidene difluoride (PVDF) membranes (Pall Australia, Victoria, Australia). The membranes were blocked and incubated with anti-Runx-2, anti-BMP-2, and anti-TXNIP mAbs (Sigma-Aldrich, New South Wales, Australia); an anti-β-actin mAb (Biosharp, Beijing, China) was used as the internal control. The membranes were exposed to a horseradish peroxidase-conjugated secondary antibody and protein bands were detected by enhanced chemiluminescence (Biosharp, Beijing, China).

### Mineralization assay

The extent of matrix mineralization in cultured VSMCs was assayed by Alizarin Red S staining. Cells were fixed in 4% formaldehyde for 10 min at room temperature, exposed to 2% Alizarin Red S for 30 min, and washed in PBS to remove excess dye.

### Measurement of alkaline phosphatase activity

The Alkaline Phosphatase Assay Kit (Beyotime, China) was applied for ALP activity assay, cells were lysed by RIPA Lysis Buffer, the lysis supernatant was used for continue assay according to the manufacturer's protocols.

### Measurement of ROS levels

ROS levels were assayed using the ROS Fluorescent Probe-DHE (Keygen, China) according to the manufacturer's protocol. Briefly, cells were incubated with 10 μM dihydroethidium in serum-free DMEM at 37°C for 20 min. The fluorescence signal was observed under a fluorescence microscope or measured using a spectrofluorophotometer at an excitation wavelength of 518 nm and an emission wavelength of 605 nm.

### Measurement of superoxide dismutase activity and MDA concentration

SOD activity and MDA concentration were measured by Total Superoxide Dismutase Assay Kit with WST-1 (Beyotime, China) and Lipid Peroxidation MDA Assay Kit (Beyotime, China), respectively. According to the manufacturer's protocols. Briefly, cells were washed twice with PBS then lysed by RIPA Lysis Buffer, cell debris were deleted through centrifuge, and the supernatant was used for continue assay. The absorbance of all samples was measured using a microplate reader (SOD, 450 nm; MDA, 530 nm) (6).

### miRNA target prediction and luciferase reporter assay

miR-146a target genes were identified using miRDB. To assess the direct binding of miR-146a to *TXNIP*, a luciferase reporter assay was performed. The entire 3′-UTR of human *TXNIP* was cloned and inserted downstream of a p-miR-reporter plasmid (Genomeditech, Shanghai, China). To assess binding specificity, the sequences that interacted with the miR-146a seed sequence were mutated at both binding positions.

### Statistical analyses

All data are presented as the mean ± standard deviation (SD) of three independent experiments. Data were analyzed and plotted using GraphPad Prism software. One-way ANOVA followed by the Bonferroni test was used to compare multiple groups, and Student's *t*-test was used to compare the means of two groups. A *p* < 0.05 was considered to be statistically significant.

## Results

### Effect of AGEs-BSA on the viability of VSMCs

**Figure 1:**
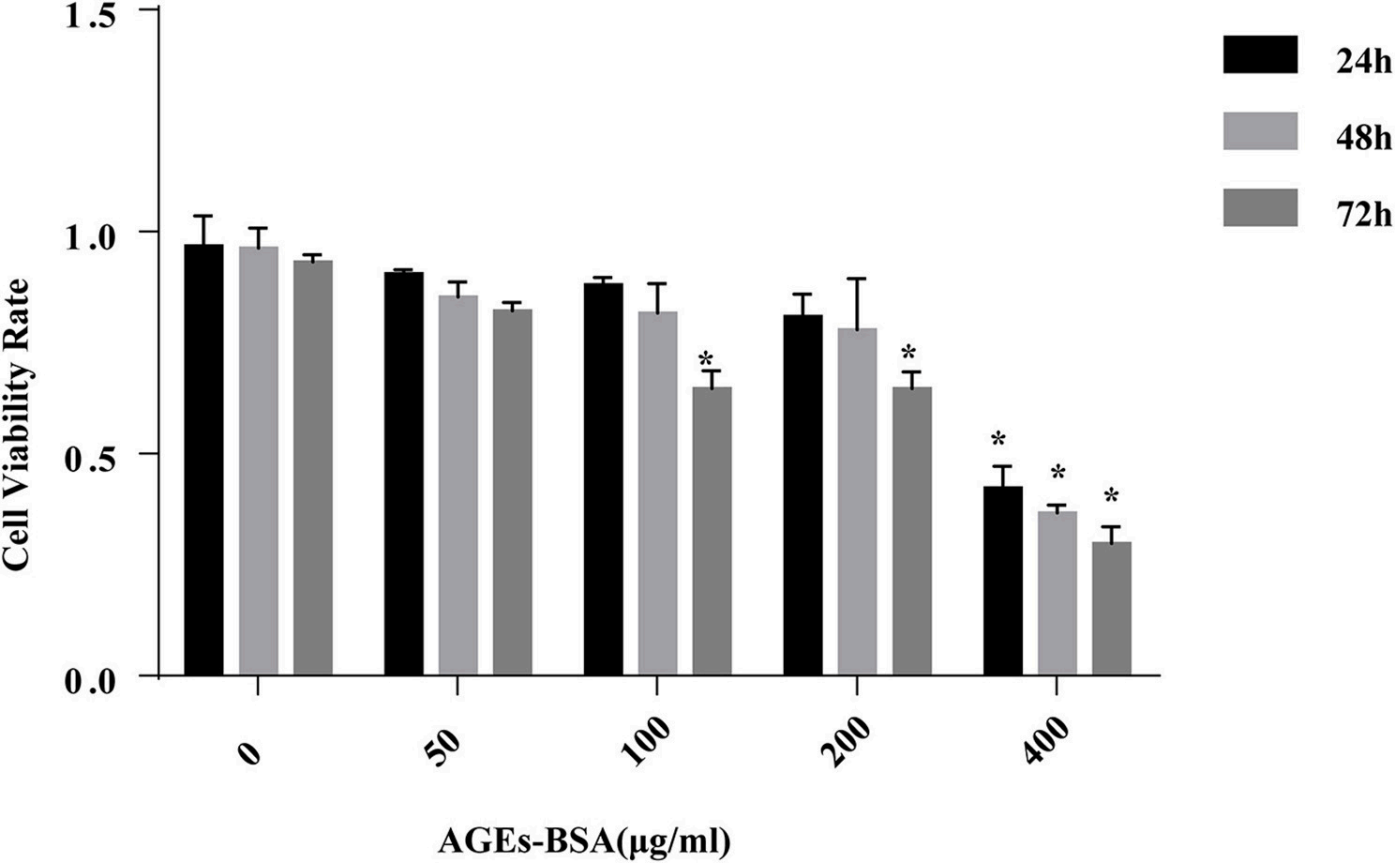
Effects of AGEs-BSA on the viability of VSMCs. VSMCs were incubated with AGEs-BSA (0, 50, 100, 200, or 400 μg/mL) for 24–72 h. The percentage of viable cells was normalized to that of the control. ^*^p < 0.05 compared to the controls.

Incubation of VSMCs with 0–200 μg/mL AGEs-BSA for 48 h did not influence their viability, but 400 μg/mL AGEs-BSA reduced the viability of VSMCs in a time-dependent manner (Figure 1). Thus, 0–200 μg/mL AGEs-BSA and incubation for 0–48 h were applied in subsequent experiments.

### AGEs-BSA promotes the osteogenic differentiation of VSMCs

**Figure 2:**
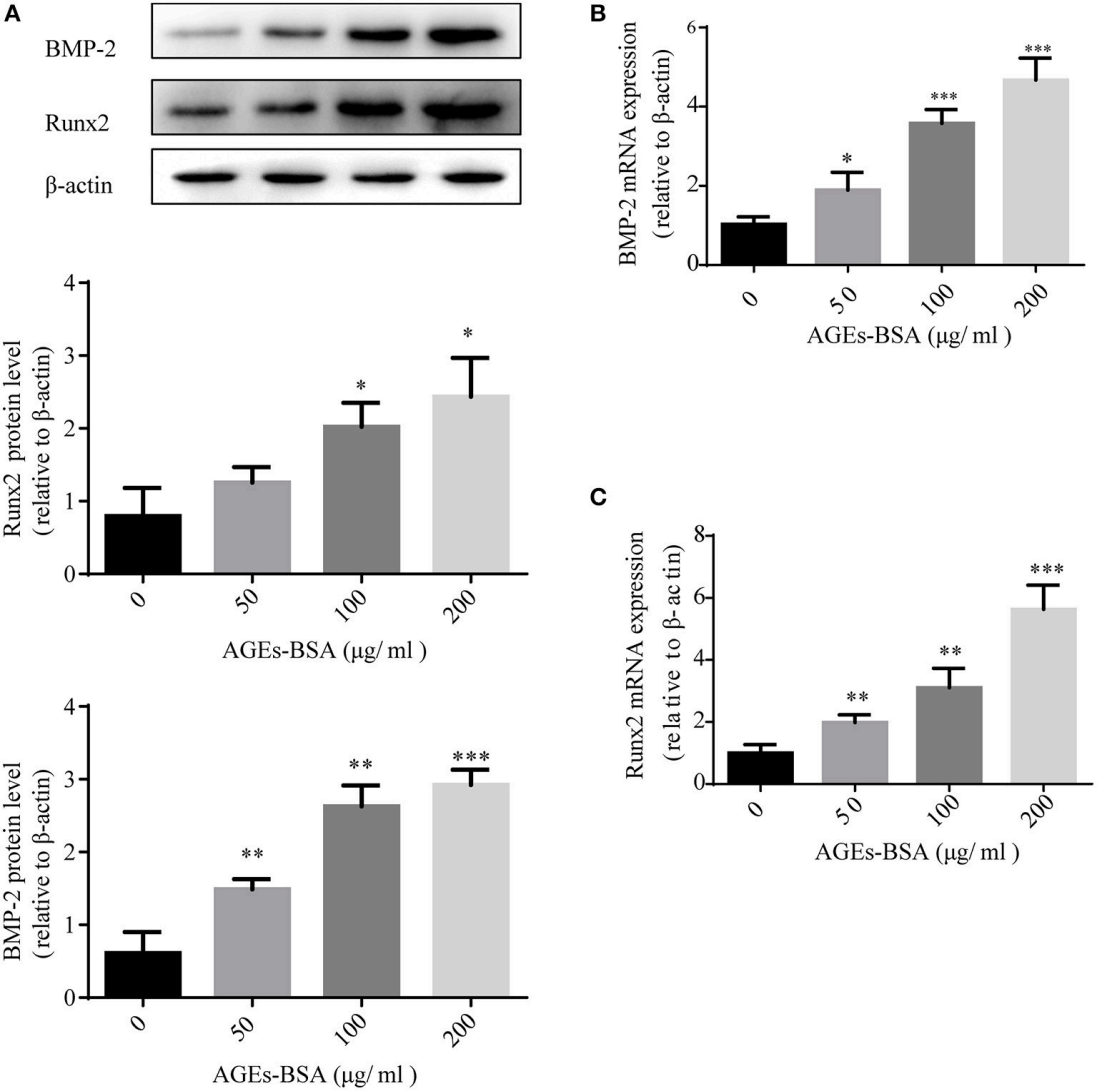
AGEs-BSA upregulates BMP-2 and Runx2 expression in VSMCs. BMP-2 and Runx2 mRNA and protein levels in VSMCs treated with 200 μg/mL BSA(Control group) or 50–200 μg/mL AGEs-BSA for 48 h. Results are means ± SD. ^*^p < 0.05, ^**^p < 0.01, and ^***^p < 0.001 vs. the Control group.

We investigated the effects of AGEs-BSA on the osteogenic differentiation of VSMCs. Application of AGEs-BSA increased the protein and mRNA levels of BMP-2 and Runx2 (markers of osteogenic differentiation) in a dose-dependent manner (Figures 2A–C). Thus, AGEs-BSA induced the osteogenic differentiation of VSMCs.

### AGEs-BSA modulates miR-146a expression

**Figure 3:**
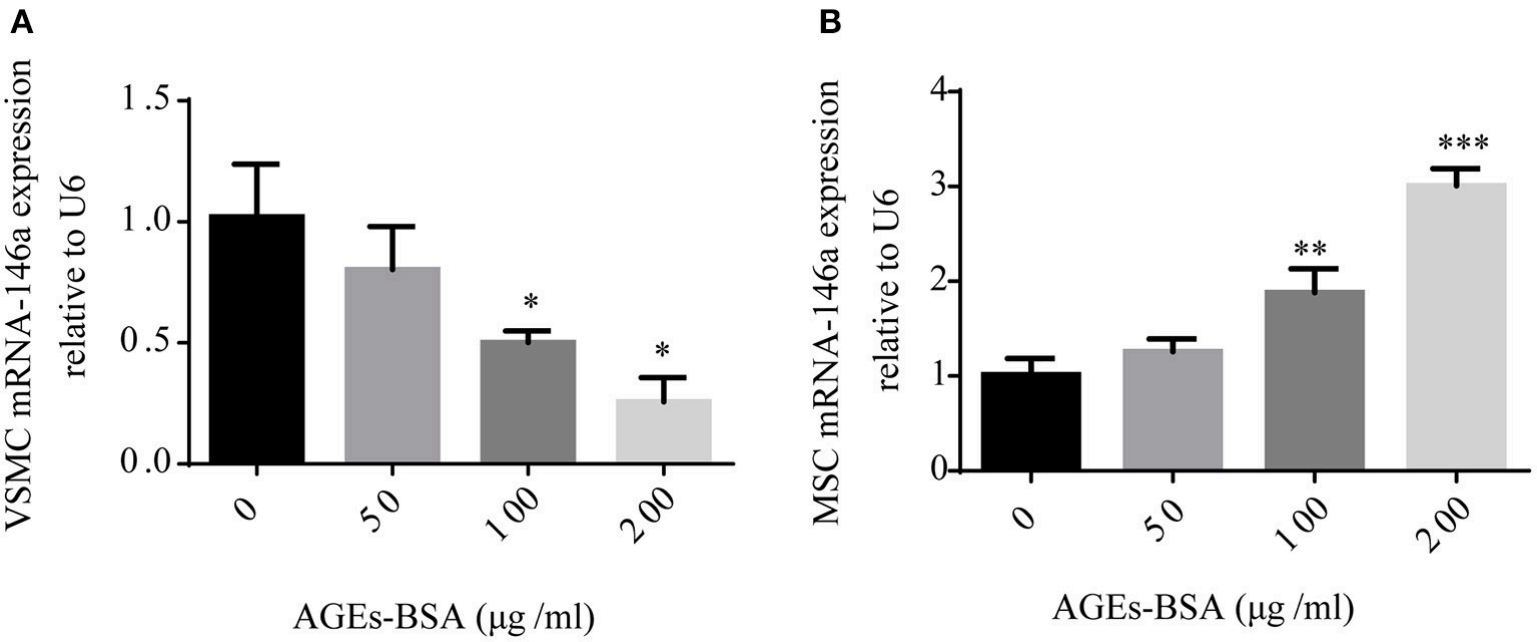
AGEs-BSA modulates miR-146a expression in MSCs and VMSCs. **(A,B)** MSCs and VSMCs were treated with 50–200 μg/mL AGEs-BSA or with 200 μg/mL BSA (Control) for 48 h, and the miR-146a level was determined by qPCR. ^*^p < 0.05, ^**^p < 0.01, and ^***^p < 0.001 vs. the control group (200 μg/mL BSA).

miR-146a is implicated in both diabetic vascular complications and osteogenic differentiation of MSCs. Because AGEs-BSA has opposite effects on the osteogenic differentiation of VSMCs and MSCs, we investigated its influence on miR-146a expression. MSCs and VSMCs were cultured with or without 50, 100, or 200 μg/mL AGEs-BSA for 48 h and miRNA-146a levels were measured using qPCR. AGEs-BSA increased and decreased miR-146a levels in MSCs and VSMCs, respectively in a dose-dependent manner (Figure 3).

### Isolation and identification of MSC-derived exosomes

**Figure 4:**
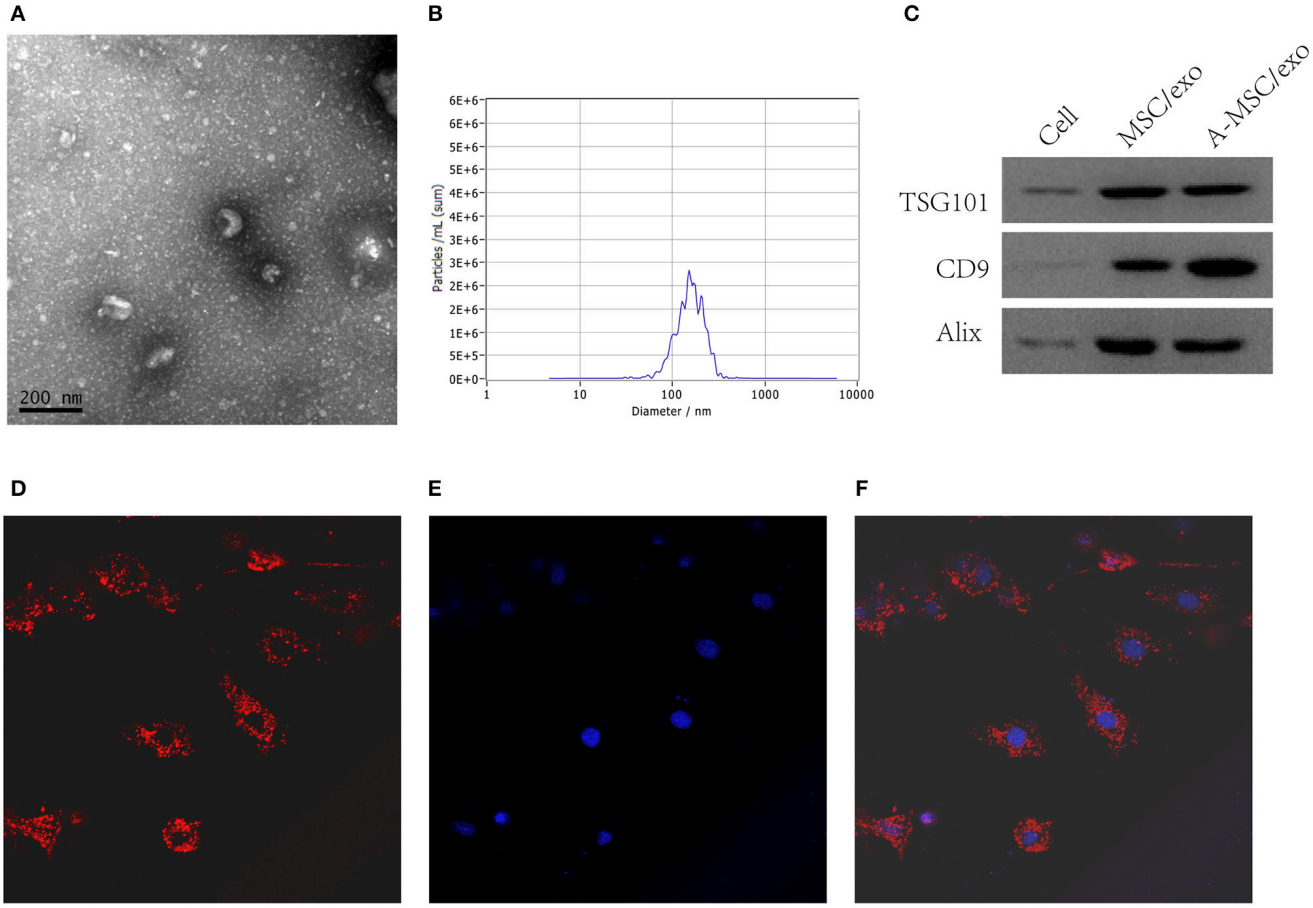
Isolation and identification of exosomes from MSCs. Exosomes were isolated from MSC culture supernatants. **(A)** TEM image of exosomes. **(B)** Nanosight analysis for the particle size. **(C)** Detection of CD9, TSG101, and Alix by Western blotting. **(D)** Confocal scanning laser microscopy showing PKH-26-labeled exosomes internalized by VSMCs (200×). **(E)** DAPI. **(F)** Merge.

By transmission electron microscopy, all exosomes were spherical and of 50–100 nm diameter (Figure 4A). Nanosight analysis demonstrated that the size of exosomes were distributed among 50–200 nm (Figure 4B).The purified exosomes were positive for the exosome markers CD9, TSG101, and Alix (Figure 4C). Moreover, confocal scanning laser microscopy showed that PKH26-labeled exosomes were internalized by VSMCs after incubation for 24 h (Figures 4D–F).

### MSCs pretreated with AGEs-BSA inhibit the calcification of VSMCs in a manner dependent on exosome transfer

**Figure 5:**
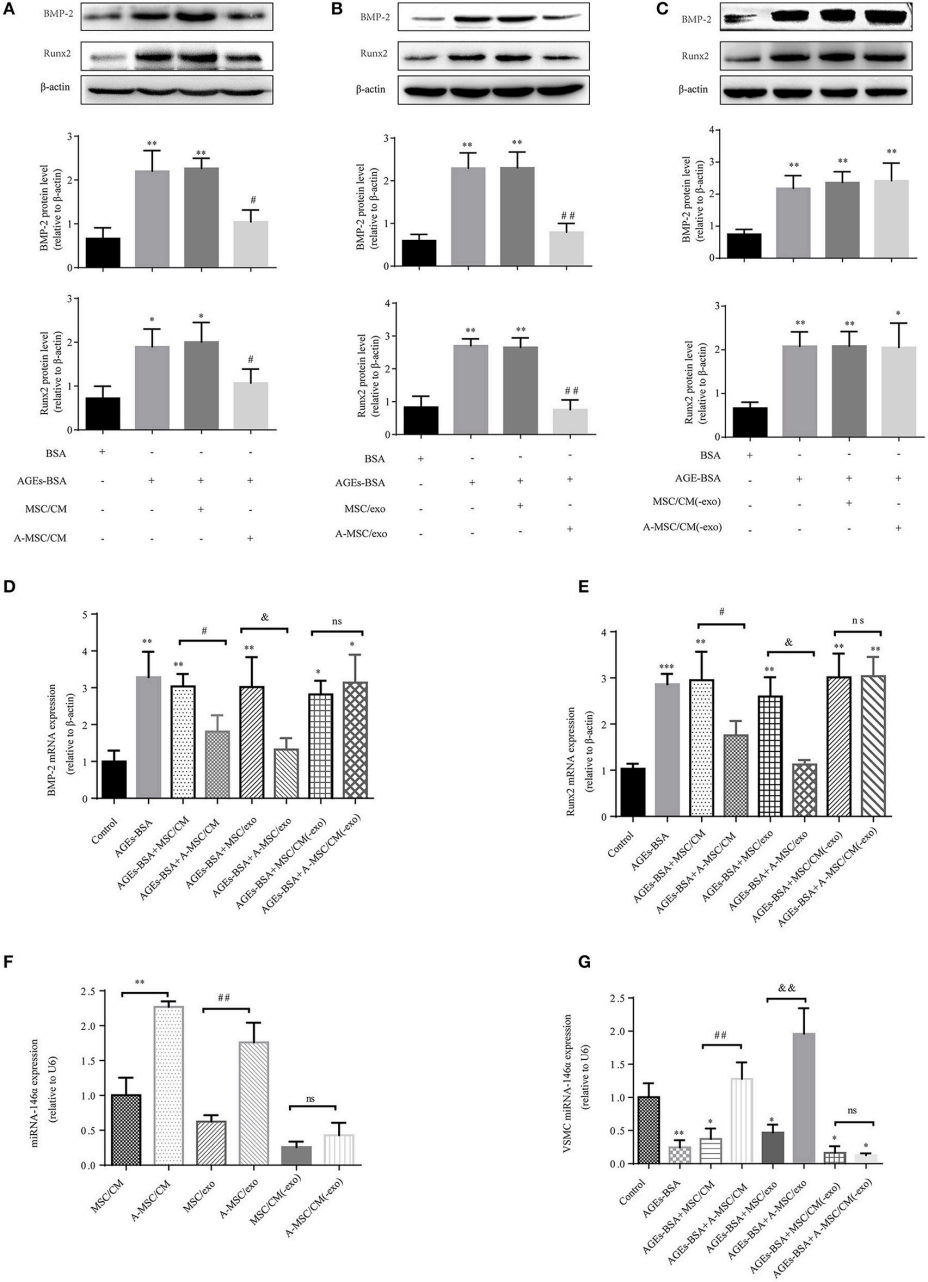
MSCs pretreated with AGEs-BSA transfer miR-146a in exosomes to inhibit osteogenic differentiation of VSMC. VSMCs were cultured in 200 μg/mL BSA, AGEs-BSA, AGEs-BSA+MSC/CM, AGEs-BSA+A-MSC/CM, AGEs-BSA+MSC/exo, AGEs-BSA+A-MSC/exo, AGEs-BSA + MSC/CM(-exo), or AGEs-BSA +A-MSC/CM(-exo). **(A–C)** Runx2 and BMP-2 protein levels determined by Western blotting. **(D,E)** Runx2 and BMP-2 mRNA levels were determined by qPCR. ^*^p < 0.05, ^**^p < 0.01, and ^***^p < 0.001 vs. the control group (200 μg/mL BSA);^#^*p* < 0.05 AGEs-BSA+MSC/CM vs. AGEs-BSA+A-MSC/CM; ^&^*p* < 0.05 AGEs-BSA+MSC/exo vs. AGEs-BSA+A-MSC/exo; ^ns^*p* > 0.05 AGEs-BSA+MSC/CM(-exo) vs. AGEs-BSA+A-MSC/CM(-exo). **(F)** miR-146a level determined by qPCR. ^**^p < 0.01 MSC/CM vs. A-MSC/CM; ^##^*p* < 0.01 MSC/exo vs. A-MSC/exo; and ^ns^*p* > 0.05 MSC/CM(-exo) vs. A-MSC/CM(-exo). **(G)** miR-146a level in VSMCs determined by RT-qPCR. ^*^p < 0.05 and ^**^p < 0.01 vs. the control group (200 μg/mL BSA); ^##^*p* < 0.01 AGEs-BSA+MSC/CM vs. AGEs-BSA+A-MSC/CM; ^&&^*p* < 0.01 AGEs-BSA+MSC/exo vs. AGEs-BSA+A-MSC/exo; and ^ns^*p* > 0.05 AGEs-BSA+MSC/CM(-exo) vs. AGEs-BSA+A-MSC/CM(-exo).

Condition medium (CM) of MSCs cultured in DMEM with and without 200 μg/mL AGEs-BSA for 48 h are termed MSC/CM and A-MSC/CM, respectively; exosomes isolated from MSC/CM or A-MSC/CM are termed MSC/exo and A-MSC/exo respectively; exome-free supernatants are termed MSC/CM(-exo) and A-MSC/CM(-exo). In cultured VSMCs treated with AGEs-BSA, administration of MSC/CM or MSC/exo had little effect on the AGEs-BSA-induced calcification. By contrast, A-MSC/CM and A-MSC/exo significantly reduced Runx2 and BMP-2 expression; this effect was reversed by exosome depletion (Figures 5A–E). Therefore, exosomes from MSCs pretreated with AGEs-BSA induce the osteogenic differentiation of VSMCs. The miR-146a levels in MSC/CM, A-MSC/CM, MSC/exo, A-MSC/exo, MSC/CM(-exo), and A-MSC/CM(-exo) were determined, and their effects on the miR-146a level in VSMCs were evaluated. AGEs-BSA significantly increased the miR-146a level in A-MSC/CM and A-MSC/exo (Figure 5F), and miR-146a expression in recipient VSMCs was significantly upregulated after coculture with A-MSC/CM and after transfer of A-MSCs–derived exosomes; the other treatments had little effect (Figure 5G). Therefore, exosomal miR-146a is involved in inhibition of the calcification of VSMCs.

### MSC-derived miR-146a-containing exosomes inhibit osteogenic differentiation of VSMC

**Figure 6:**
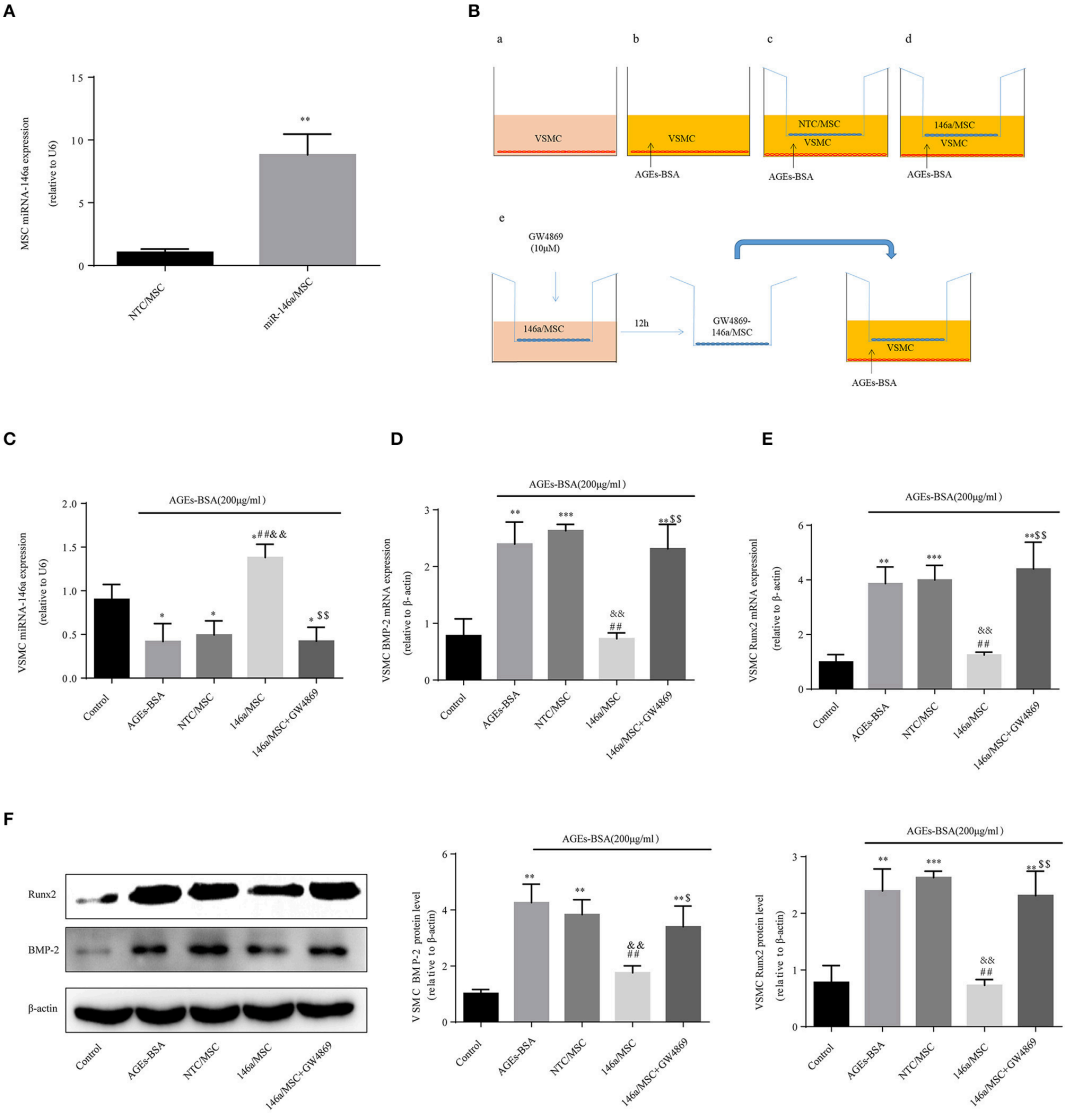
MSC-derived miR-146a-containing exosomes inhibit the osteogenic differentiation of VSMCs. **(A)** MSCs were transfected with a miR-146a-expressing plasmid, and the transfection efficiency was determined by qPCR. ^**^p < 0.015 vs. NTC/MSC group. **(B)** The Transwell system. **(C)** miR-146a expression determined by qPCR. ^*^p < 0.05 vs. the control group; ^##^*p* < 0.01 vs. AGEs-BSA group; ^&&^*p* < 0.01 vs. AGEs-BSA+NTC/MSC group; and ^$$^*p* < 0.01 vs. AGEs-BSA+146a/MSC group. **(D,E)** Runx2 and BMP-2 mRNA levels were determined by qPCR. **(F)** Runx2 and BMP-2 protein levels determined by Western blotting. ^*^p < 0.05, ^**^p < 0.01, and ^***^p < 0.001 *vs*. the control group; ^##^*p* < 0.01 vs. AGEs-BSA group; ^&&^*p* < 0.01 vs. AGEs-BSA+NTC/MSC group; ^$$^*p* < 0.01 vs. AGEs-BSA+146a/MSC group.

Then miR-146a was overexpressed in MSCs, which were transfected for 48 h with pre-miR-146a (miR-146a/MSCs) or a plasmid harboring an None-Target Control sequence (NTC/MSCs). The miR-146a expression level in MSCs was 8.8-fold higher than that in NTC/MSCs (Figure 6A). VSMCs, with or without addition of AGEs-BSA, were cocultured for 48 h in Transwells with miR-146a/MSCs or NTC/MSCs (Figure 6B), and transfer of miR-146a was confirmed by qPCR. The miR-146a expression was significantly higher in VSMCs cocultured with miR-146a/MSCs than in NTC-MSCs (Figure 6C). Next, miR-146a/MSCs were treated with the exosome-release inhibitor GW4869 for 12 h before coculture (Figure 6B). GW4869 significantly decreased the miR-146a level in VSMCs in the 146a/MSC+GW4869 group compared to the 146a/MSC group (Figure 6C). Thus, miR-146a is transferred from MSCs to VSMCs in exosomes. The effects of MSC-derived miR-146a on Runx2 and BMP-2 expression in VSMCs were next assessed. AGEs-BSA induced a significant increase in Runx2 and BMP-2 expression in VSMCs compared to BSA. Coculture of AGEs-BSA-treated VSMCs with miR-146a/MSCs significantly reduced the Runx2 and BMP-2 mRNA (Figures 6D,E) and protein (Figure 6F) levels compared to those in GW4869-pretreated VSMCs cocultured with miR-146a/MSCs. Therefore, pretreatment of MSCs with AGEs-BSA inhibits the calcification of VSMCs in a manner dependent on exosomes.

### Purified 146a/exo inhibit the calcification of VSMCs

**Figure 7:**
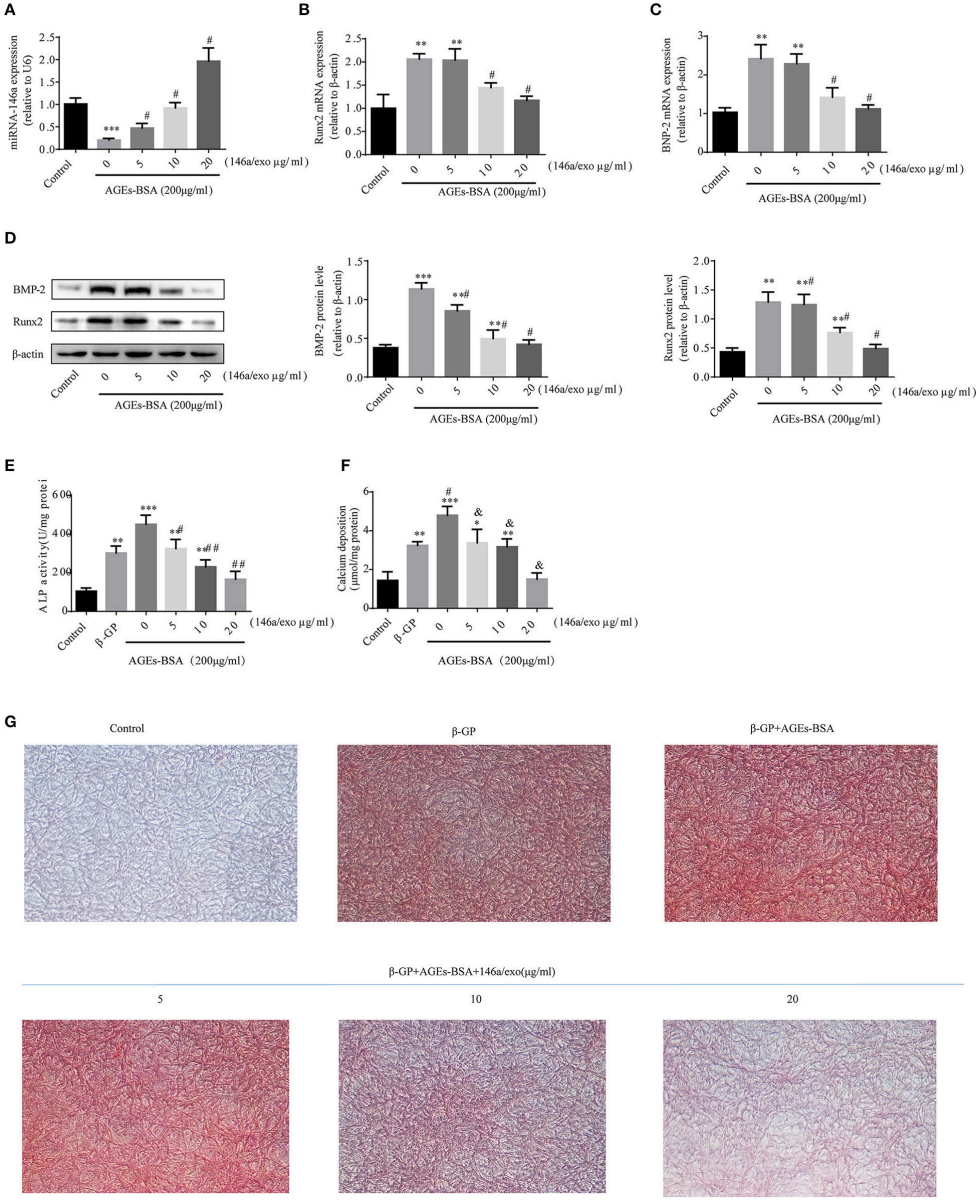
146a/exo inhibits AGEs-BSA-induced calcification of VSMC. **(A)** miR-146a expression determined by qPCR. ^*^p < 0.05, ^**^p < 0.01, and ^***^p < 0.001 vs. the control group and; ^#^*p* < 0.05 vs. AGEs-BSA group. Protein **(B)** and mRNA **(C,D)** levels of Runx2 and BMP-2 determined by Western blotting and qPCR. ^*^p < 0.05, ^**^p < 0.01, and ^***^p < 0.001 vs. the control group and ^#^*p* < 0.05 vs. AGEs-BSA group. **(E)** ALP activity. ^*^p < 0.05 vs. the control group; ^#^*p* < 0.05 vs. β-GP group; and ^&^*p* < 0.05 vs. β-GP +AGEs-BSA group. **(F)** Intracellular calcium content. ^*^p < 0.05 vs. the control group; ^#^*p* < 0.05 vs. β-GP group; and ^&^*p* < 0.05 *vs*. β-GP +AGEs-BSA group. **(G)** Calcium deposition by Alizarin Red S staining (magnification, 100×).

Treatment of VSMCs with 200 μg/mL AGEs-BSA for 48 h decreased miR-146a expression, and this effect was prevented by miRNA-146a–containing exosomes (Figure 7A). Moreover, 5–20 μg/mL 146a/exo inhibited the AGEs-BSA-induced upregulation of Runx2 and BMP-2 Mrna (Figures 7B,C) and protein (Figure 7D) levels in VSMCs in a dose—dependent manner. To investigate the effects of 146a/exo on AGEs-BSA-induced calcification, VSMCs were cultured in the presence of 10 mM β-GP, 200 μg/mL AGEs-BSA, and 0, 5, 10, or 20 μg/mL 146a/exo for 7 or 21 days. ALP activity was significantly upregulated by AGEs-BSA on day 7 (Figure 7E) but was inhibited by miR-146a in a dose-dependent manner. Application of AGEs-BSA markedly increased the intracellular calcium concentration and calcium deposition in VSMCs on day 21, while 146a/exo significantly decreased the intracellular calcium content (Figure 7F) and the formation of calcified deposits (Figure 7G) in VSMCs.

### Effects of AGEs-BSA on TXNIP and ROS production

**Figure 8:**
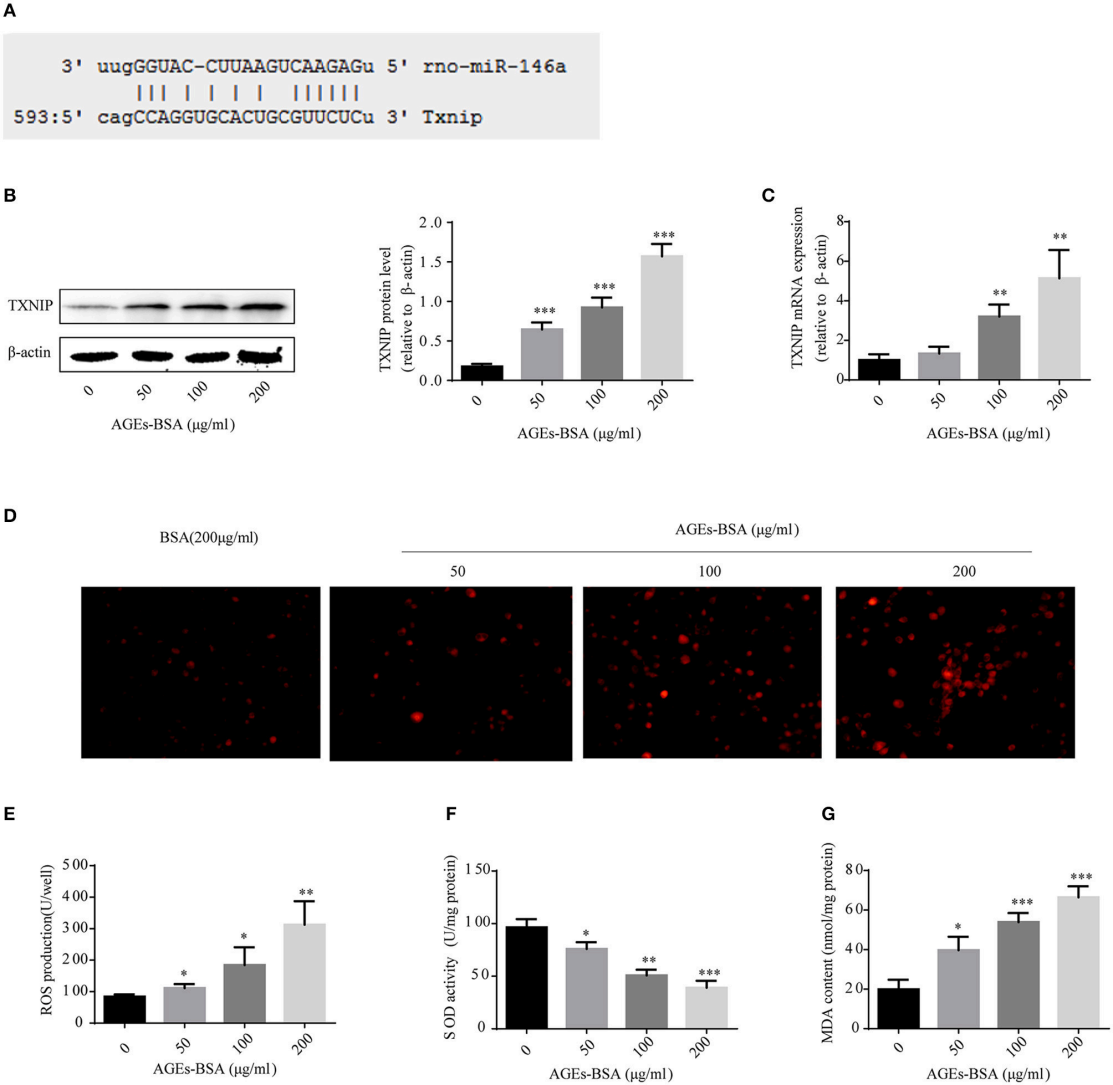
Effects of AGEs-BSA on *TXNIP* expression and ROS levels. **(A)** The predicted miR-146a binding sites within 3′ UTR of TXNIP. **(B,C)** TXNIP protein and mRNA levels determined by Western blotting and qPCR. **(D)** ROS concentrations and fluorescence micrographs of ROS levels. **(E)** ROS production. **(F)** SOD activity. **(G)** MDA content. ^*^p < 0.05, ^**^p < 0.01, and ^***^p < 0.001 vs. the control group.

To identify genes whose expression was impacted by 146a/exo, we performed computational prediction using a miRNA target prediction program (miRDB). TXNIP was predicted to be a target of miR-146 (Figure 8A). TXNIP is implicated in diabetic oxidative stress injury (39–41), and the up- and down-regulation of TXNIP is significantly associated with cell oxidative stress level in VSMCs (Supplementary Figure 2). What's more, oxidative stress is an important pathological mechanism of AGEs-BSA-induced VC. Therefore, we determined whether the AGEs-BSA-mediated decrease in miR-146a level is associated with upregulation of TXNIP expression and increased ROS production in VSMCs. TXNIP was upregulated by AGEs-BSA in a dose-dependent manner (Figures 8B,C), which correlated with intracellular ROS production (Figures 8D,E). SOD activity was significantly decreased (Figure 8F), while MDA content was significantly increased by AGEs-BSA treatment compared to the control (Figure 8G).

### Effects of miR-146a/exo on AGEs-BSA-induced VSMCs TXNIP expression and ROS production

**Figure 9:**
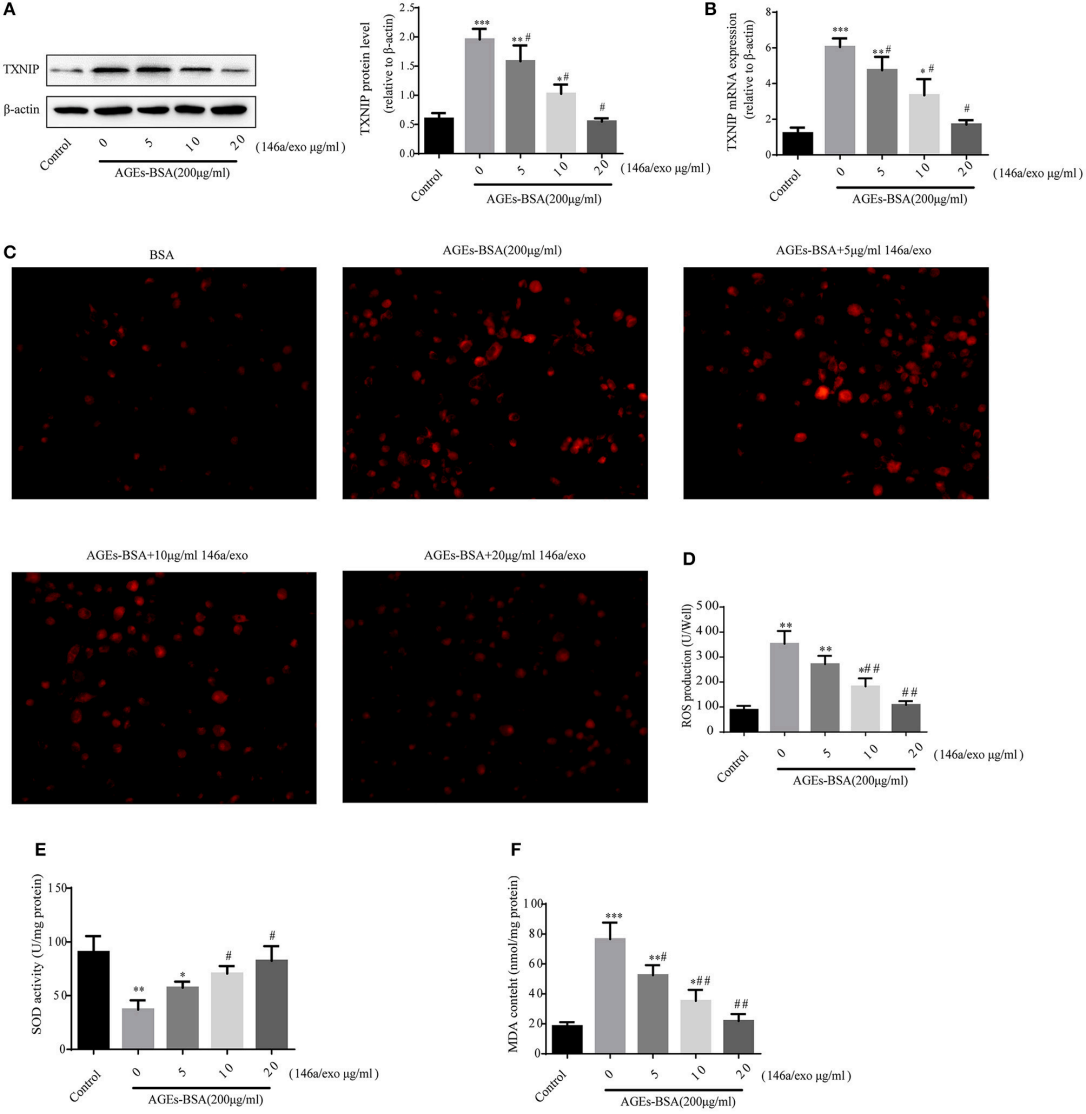
Effects of miR-146a/exo on AGEs-BSA–induced *TXNIP* expression and ROS production in VSMCs. **(A,B)** TXNIP mRNA and protein levels by Western blotting and qPCR. **(C)** ROS concentrations and fluorescence micrographs of ROS levels. **(E)** ROS production. **(F)** SOD activity. **(G)** MDA content. ^*^p < 0.05, ^**^ *p* < 0.01, and ^***^ *p* < 0.001 vs. the control group; ^#^*p* < 0.05 and ^##^*p* < 0.01 vs. AGEs-BSA group.

Treatment of VSMCs with 200 μg/mL AGEs-BSA and 0–20 μg/mL 146a/exo for 48 h decreased the TXNIP protein and mRNA levels (Figures 9A,B), reduced ROS (Figures 9C,D), and MDA (Figure 9F) production, and increased SOD activity (Figure 9E) compared to none-146a/exo treatment.

### TXNIP overexpression reverses the protective effects of 146a/exo

**Figure 10:**
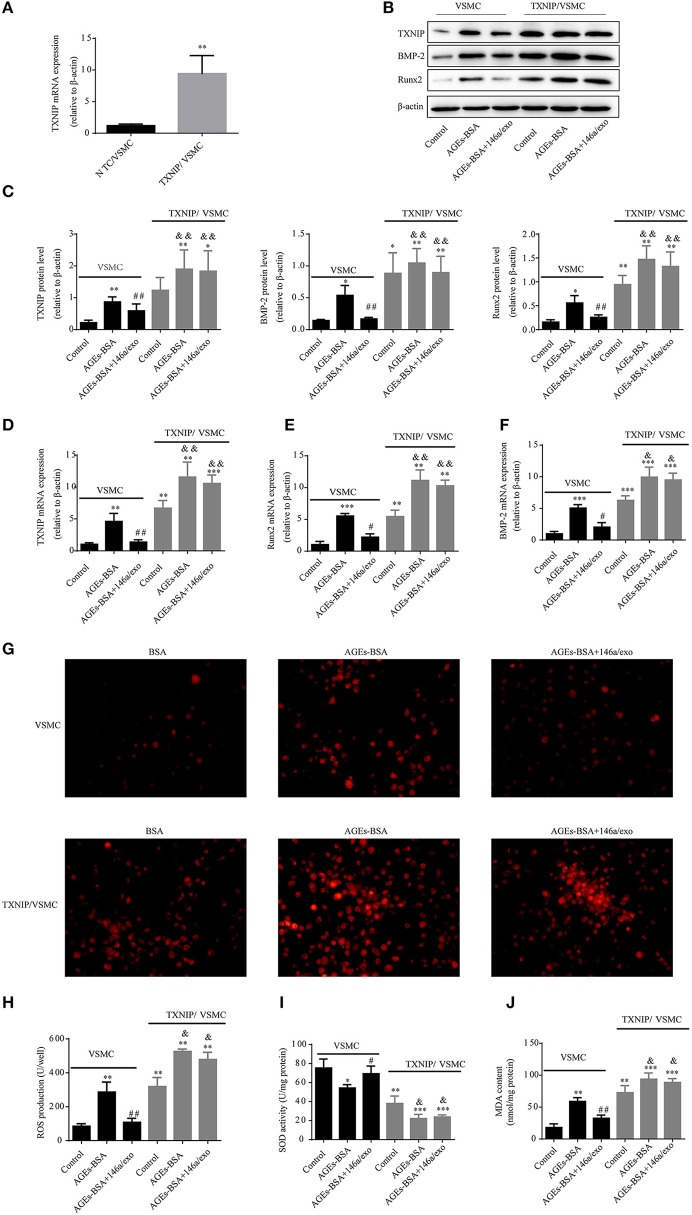
TXNIP overexpression reverses the protective effects of 146a/exo. **(A)** Confirmation of transfection of VSMCs with a TXNIP-expressing plasmid by qPCR. **(B,C)** TXNIP, Runx-2, and BMP-2 protein levels by Western blotting. **(D–F)** *TXNIP, Runx-2*, and *BMP-2* mRNA levels by qPCR. **(G)** ROS concentrations and fluorescence micrographs of ROS levels. **(H)** ROS production. **(I)** SOD activity. **(J)** MDA content. ^*^p < 0.05, ^**^p < 0.01, and ^***^p < 0.001 vs. VSMC control group; ^#^*p* < 0.05 and ^##^*p* < 0.01 vs. VSMC AGEs-BSA group; and ^&^*p* < 0.05 and ^&&^*p* < 0.01 vs. TXNIP/VSMC control group.

TXNIP overexpression counteracted the anti-oxidative stress and anti-calcification effects of miR-146a (Figure 10). Therefore, AGEs-BSA-mediated induction of TXNIP expression is involved in generation of oxidative stress, while 146a/exo inhibits *TXNIP* expression and reduces the level of oxidative stress.

### TXNIP 3′UTR is targeted by miR-146a

**Figure 11:**
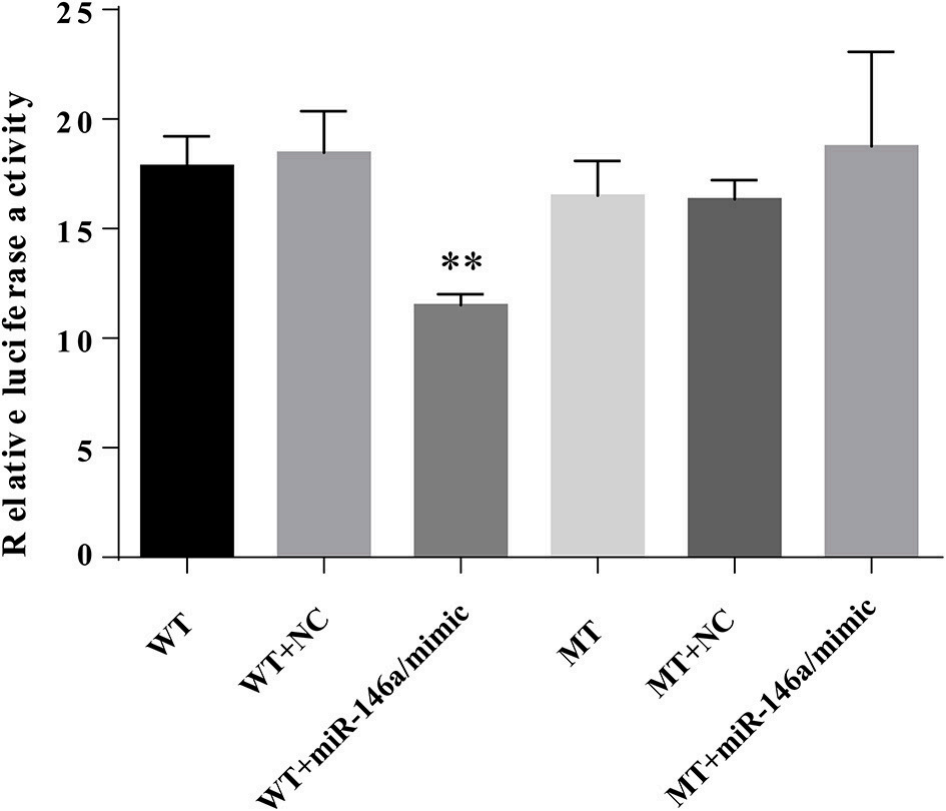
miR-146a directly targets TXNIP 3′UTR. VSMCs were transiently co-transfected with PGL3-CMV-LUC-MCS-TXNIP−3′UTR dual luciferase reporter recombinant vector, and miR-146a mimic or NTC mimic. The firefly luciferase activity was detected by Dual Luciferase Reporter Gene Assay Kit.

To verify the interaction between miR-146a and the 3′UTR of TXNIP, luciferase reporter assays were performed using the wild-type TXNIP 3′UTR sequence containing either the seed sequence for miR-146a recognition (TXNIP-3′UTRwt) or a mutated 3′UTR (TXNIP-3′UTRmut). The relative luciferase activity of the TXNIP-3′UTRwt reporter was significantly reduced by miR-146a mimics compared to NC mimics; however, mutation of the miR-146a seed sequence abolished this inhibitory effect. miR-146a mimics and inhibitors decreased and increased, respectively, the TXNIP protein level (Figure 11). Therefore, the 3′UTR of TXNIP contained the direct binding site of miR-146a, indicating that miR-146a can target TXNIP and function as an inhibited factor.

### miR-146a-containing exosomes inhibit high glucose induced osteogenic differentiation of VSMC

AGEs and their effects on Vascular Biology especially in DM have received much attention, where the glucose levels are generally elevated. To clarify whether 146a/exo would be still efficient under high glucose condition. We carry out investigations by holding the VSMCs under normal and elevated glucose (25 mmol/L) level conditions in culture. As the results shown, HG upregulated the gene and protein expression of Runx2 and BMP-2 of VSMC, the TXNIP expression(Supplementary Figures 3A–E) as well as the oxidative stress level (Supplementary Figures 3F–I) were also upregulated, what's more, HG stimulated calcium deposition (Supplementary Figures 3J,K) and ALP activity (Supplementary Figure 3L) in VSMCs. These results suggest that HG accelerates VSMC calcification. Then, to determine whether 146a/exo can alleviate calcium deposition in VSMCs, VSMCs were co-incubated with the 146a/exo or with NTC/exo. We observed that treatment with 146a/exo inhibited the HG-induced VSMC calcification. Taken together, our data suggest that 146a/exo plays an important role in diabetic vascular calcification.

## Discussion

Our findings suggest that AGEs-BSA accelerate calcium deposition and increase ROS production in VSMCs. Furthermore, the expression of miR-146a, which is reportedly downregulated in patients with T2DM, was decreased in VSMCs by AGEs-BSA treatment. By contrast, AGEs-BSA upregulated the expression of miR-146a in MSCs. Moreover, A-MSC/CM and A-MSCs-derived exosomes significantly reduced the AGEs-BSA-induced expression of *Runx2* and *BMP-2* in VSMCs, whereas exosome depletion reversed the protective effects of conditioned medium. Thus, exosomes play an important role in the anti-osteogenic effects of A-MSC/CM. VSMCs cocultured with 146a/MSCs exhibited higher miR-146a levels and reduced expression of calcification-related genes; these effects of 146a/MSCs was diminished by the exosome-release inhibitor, GW4869. Therefore 146a/MSCs secrete miR-146a-containing exosomes, which inhibited the calcification of VSMCs. Indeed, 0–20 μg/mL 146a/exo inhibited the AGEs-BSA-induced increase in *TXNIP* expression in VSMCs in a dose-dependent manner, accompanied by decreased expression of osteogenic genes and production of ROS. Furthermore, overexpression of *TXNIP* in VSMCs reversed the protective effects of 146a/exo. Therefore, 146a/exo protected VSMCs against AGEs-BSA induced calcification, in part by targeting the TXNIP/ROS pathway.

VC is a form of heterotopic calcification common in patients with diabetes (42), and osteoblast-like differentiation of VSMCs is a major pathogenetic mechanism of VC (43). AGEs levels, which are increased in diabetes (44), promote VC. AGEs-induced ROS generation plays an important role in calcium deposition in VSMCs (45, 46). Therefore, further studies should focus on developing methods for inhibiting ROS production.

miR-146a is a causal factor in several chronic diseases, and reduced miR-146a expression is associated with disturbed proinflammatory cytokine levels in patients with T2DM (47). Polymorphisms in the miR-146a gene correlated with an increased risk of T1DM (48). In addition, miR-146a is consistently dysregulated in T1DM patients (49). In mice with T2DM, miR-146a mimics significantly ameliorated peripheral neuropathy, principally by suppressing the expression of hyperglycemia-induced proinflammatory genes (50). Moreover, miR-146a is reportedly involved in high-glucose-induced endothelial inflammation, which suggests that it may be a novel therapeutic target for diabetic vascular disease (25). Overexpression of miR-146a inhibits osteogenesis by MSCs (32). In this study, AGEs-BSA had the opposite effects on miR-146a expression in VSMCs and MSCs, possibly explaining its opposite effects on osteogenesis of these cells. These data suggest that inhibition of miR-146a is an important mechanism underlying AGEs-BSA-induced osteoblastic differentiation of VSMCs. Therefore, miR-146a may inhibit osteogenesis of VSMCs and MSCs, and its modulation could be used to ameliorate VC in patients with diabetes.

MSC-derived exosomes are used to treat cardiovascular disease (51–53), but their impact on VC is unclear. Although MSCs have shown promising results in chronic kidney disease-associated VC (54), while in this study, MSC-CM and exosomes did not modulate AGEs-BSA-induced VSMC calcification, possibly due to our use of different stimulatory factors and receptor cells. Preconditioned MSCs have been reported to have enhanced functionality (55–57). In this study, miR-146a expression in MSCs was upregulated by AGEs-BSA, so we speculated that the resulting miR-146a was transferred from MSCs to VSMCs through exosomes, which prevents osteogenic differentiation of VSMCs. Indeed, exosomes isolated from MSCs pretreated with AGEs-BSA had considerable inhibitory effects on the calcification of VSMCs.

Using miR-146a-overexpressing MSCs, we found that 146a/MSC coculture inhibited AGEs-BSA-induced calcification of VSMCs. Moreover, exosomes derived from 146a/MSCs contained high levels of miR-146a. In addition, PKH-26-stained exosomes were transferred to VSMCs after coculture for 24 h, resulting in an increased miR-146a level, decreased *Runx2* and *BMP-2* expression, and reduced calcification of VSMCs.

To investigate the mechanism underlying the effects of 146a/MSC-derived exosomes on the calcification of VSMCs, we searched for target genes of miR-146a in the miRDB database. During several potential targets, TXNIP, the endogenous inhibitor of ROS elimination, has attracted our attention for continue research. The ubiquitously presented thioredoxin (Trx) system is an important antioxidative mechanism which can protect cells against oxidative damage, Trx protects cells against oxidative damage (58), while TXNIP directly interacts with the catalytic center of reduced Trx and inhibits its reducing activity (59). Increased expression of TXNIP and reduced Trx activity have been observed in animal models of diabetes. TXNIP is a key factor in the regulation of functional β-cell mass, and inhibition of TXNIP ameliorates the symptoms of diabetes (40); for example, knockdown of TXNIP ameliorated high-glucose-induced epithelial—to—mesenchymal transition in renal tubular epithelial cells (60) and deletion of *TXNIP* prevented high-fat-diet-induced inflammation in critical-limb ischemic mice (61). In addition, the diabetic lipid environment can induce *TXNIP* expression, which stimulates the vascular inflammation (61). Therefore, TXNIP plays an important role in diabetic vascular complications. We found that AGEs-BSA not only induced the osteoblastic differentiation of VSMCs but also upregulated their *TXNIP* expression and ROS production. Moreover, 0–20 μg/mL 146a/exo inhibited AGEs-BSA-induced *TXNIP* expression in VSMCs in a dose-dependent manner, which was accompanied by the decreased expression of osteogenesis-related genes and ROS production. Overexpression of TXNIP in VSMCs counteracted the protective effects of 146a/exo, which suggests that 146a/exo prevents AGEs-BSA-induced calcification of VSMCs in part by targeting the TXNIP/ROS pathway. What's more, overexpression of miR-146a mimic significantly reduced the activity of firefly luciferase (FLUC) containing the 3′UTR sequence of TXNIP, indicated that TXNIP is a target gene of miR-146a in VSMC.

Despite the potential anti-inflammatory effect of miR-146a has widely been reports by the previous studies, Wei et.al reported that, miR-146a inhibits osteoblast proliferation and differentiation that results in the development of osteoporosis (32). On the other hand, the “off-target” or undesirable side effects such as osteoporosis and Vitamin D deficiency, maybe a major obstacle for future research. Therefore, it is necessary to get an ideal exosome delivering system which can deliver the miRNAs to specific cells, tissues, or organs through a “paracrine” pathway. None-systemic administration of miR-146a containing exosomes, such as local muscle injection and targeted intravascular administration, may provide a way to minimize the negative effect of 146a/exo on bone formation. What's more, we will further focus on the selective transfection of exosomes into VSMCs in future studies, such as combining exosomes with the specific receptors of VSMCs.

## Conclusions

In summary, AGEs-BSA decreased and increased the expression of miR-146a and TXNIP, respectively, and increased ROS production, in VSMCs, which promoted their osteogenic differentiation. By contrast, AGEs-BSA upregulated miR-146a expression in MSC exosomes. Therefore, exosomes derived from AGEs-BSA-pretreated or miR-146a-transfected MSCs prevent the calcification of VSMCs, partly through the negatively regulation of TXNIP gene. These results suggest that miR-146a containing exosomes may function as a potential method in VSMC calcification therapy.

## Author contributions

NL conceived the experiments. YW conceived the experiments, performed the experiments, analyzed the data, and wrote the paper. W-QM conceived the experiments, wrote the paper. YZ performed the experiments. X-QH performed the experiments.

### Conflict of interest statement

The authors declare that the research was conducted in the absence of any commercial or financial relationships that could be construed as a potential conflict of interest.

## Footnotes

^1^http://www.mirdb.org/
