# Supplementary material for: Exosomes Derived From Mesenchymal Stromal Cells Pretreated With Advanced Glycation End Product-Bovine Serum Albumin Inhibit Calcification of Vascular Smooth Muscle Cells
Source: Front Endocrinol (Lausanne). 2018 Sep 21;9:524. doi: 10.3389/fendo.2018.00524 (PMC6160580; doi:10.3389/fendo.2018.00524)
Supplement: Supplementary file 1 [file Presentation_1.pdf]

Supplementary Figure1

A

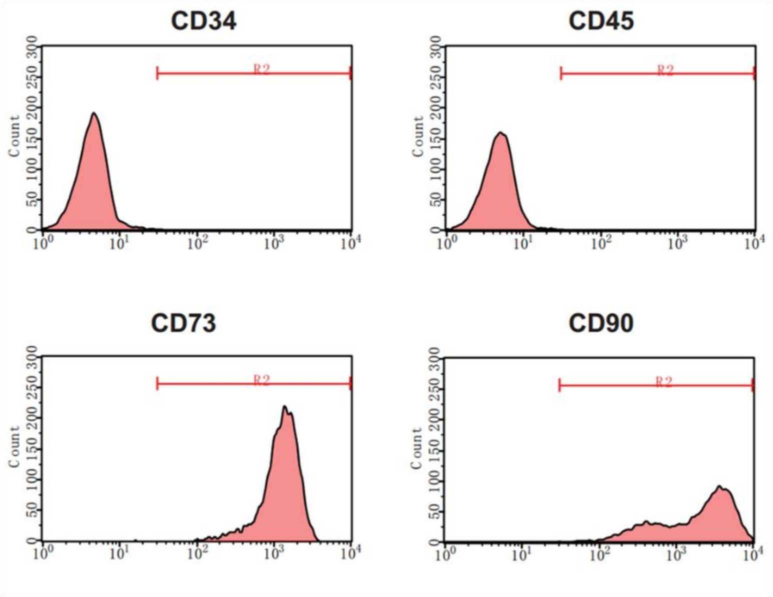

B

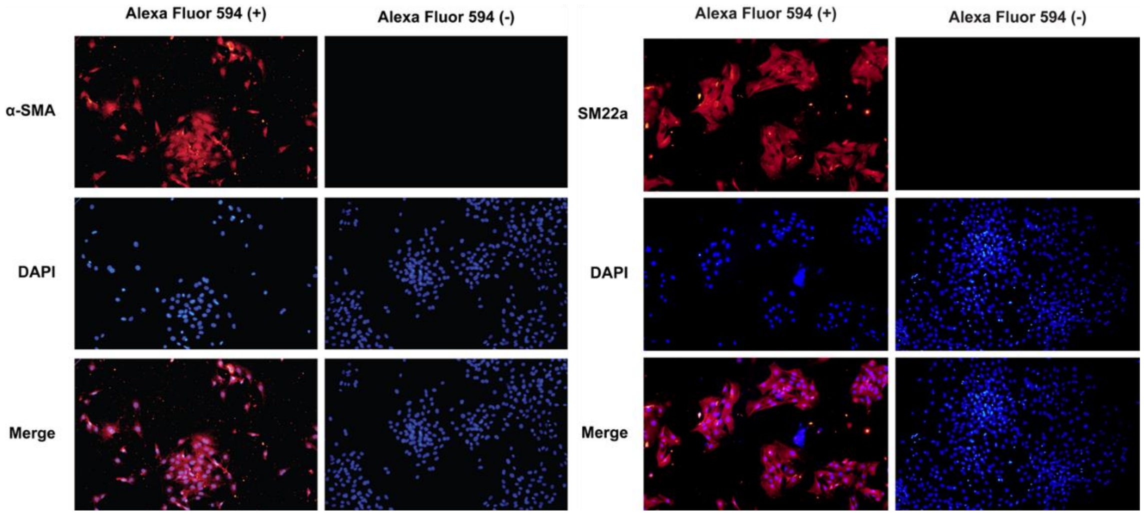

Supplementary Figure1 Identification of primary SD rat MSC and VSMC. (A) Fluorescence-activated cell sorting (FACS) analysis for MSC negative cell markers (CD34 and CD45) and the positive cell markers (CD73 and CD90). FITC: Fluorescein isothiocyanate. (B) Immunofluorescence stain for the VSMC markers (α-SMA and SM-22α).

Supplementary Figure 2

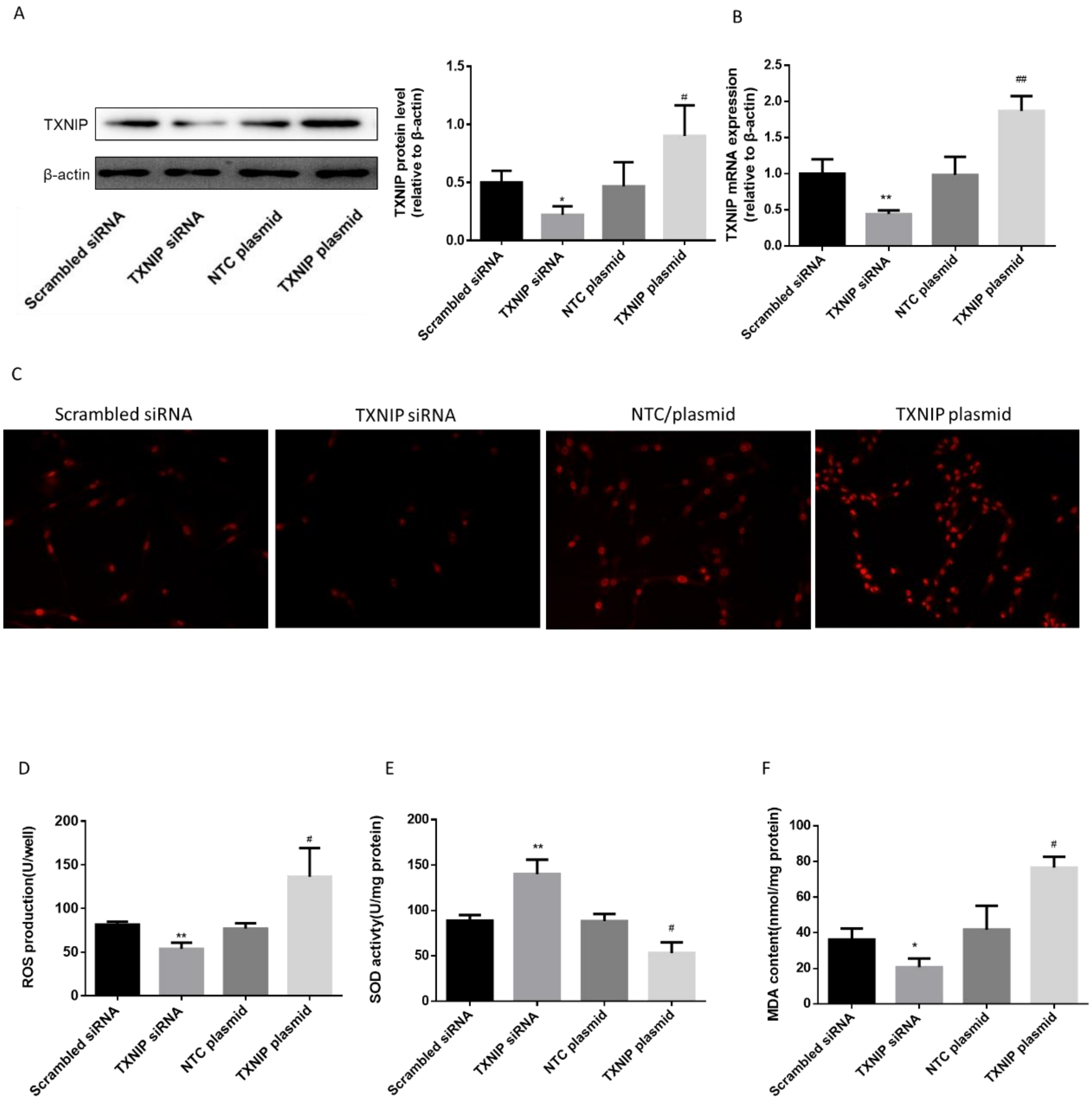

Supplementary Figure2 The effect of TXNIP on VSMC oxidative stress level.

(A)Western blot analysis for the TXNIP protein expression in VSMC after transfected by TXNIP siRNA and TXNIP-overexpressing plasmid. The Scrambled siRNA and non-target control plasmid were used as control group. (B) qPCR analysis for the TXNIP mRNA expression. (C) Fluorescent microscope image of ROS production in VSMC. (D) ROS production. (E) SOD activity. (F) MDA content. * $p < 0.05$, ** $p < 0.01$, and *** $p < 0.001$ vs. scrambled siRNA group; # $p < 0.05$ and ## $p < 0.01$ vs. NTC/plasmid group.

Supplementary Figure3

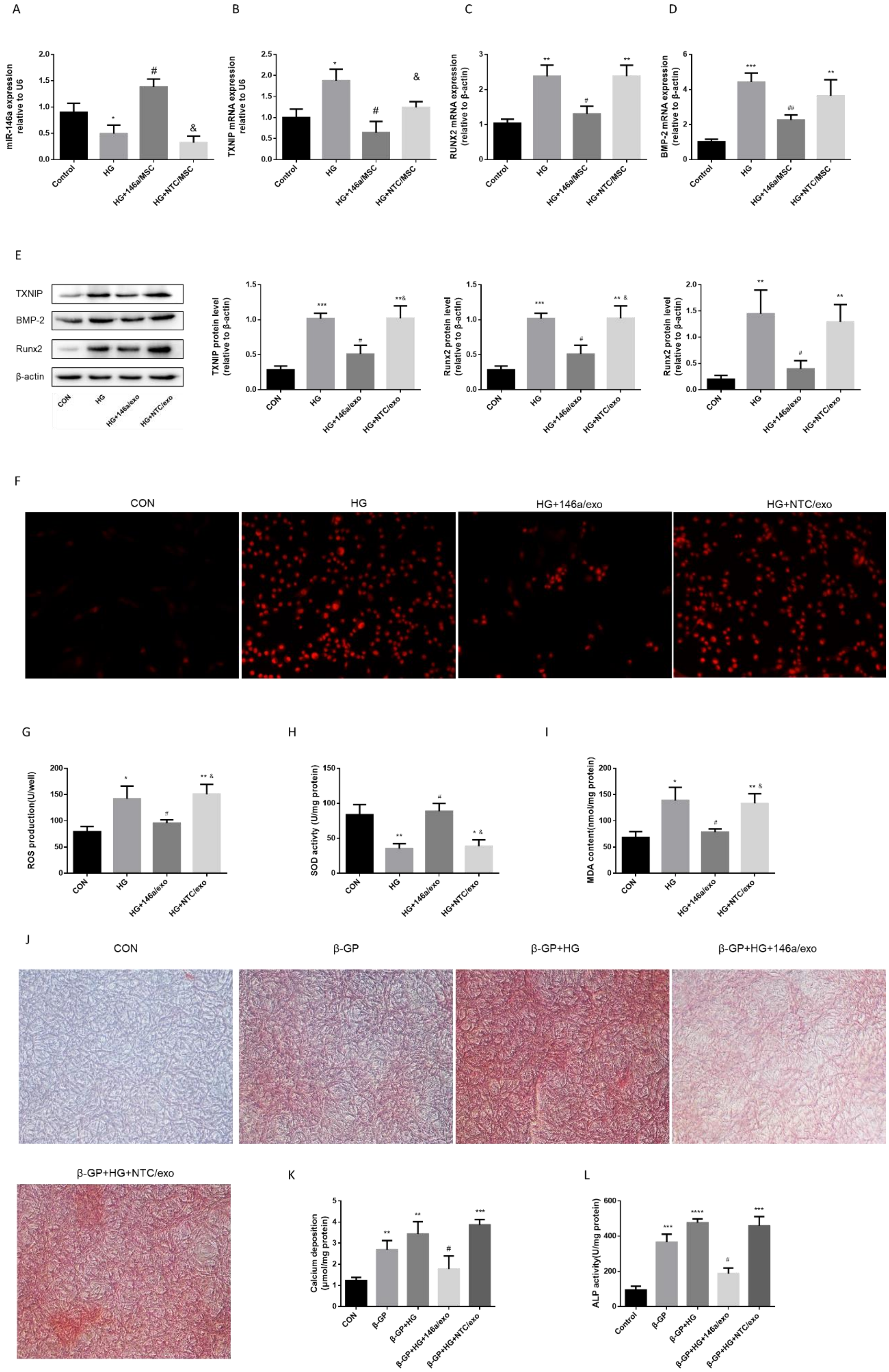

Supplementary Figure3 Effects of miR-146a-overexpresssed exosomes on HG-

induced *TXNIP* expression, ROS production and osteogenic differentiation in VSMCs

(A-D) Measurement of miR-146a, TXNIP, Runx2, and BMP-2 mRNA expression by qPCR. (E) Western blot analysis for the protein expression of TXNIP, Runx2, and BMP-2. (F) Fluorescence micrographs of ROS levels. * $p < 0.05$, ** $p < 0.01$, and *** $p < 0.001$ vs. the control group; # $p < 0.05$ and ## $p < 0.01$ vs. HG group

(G) ROS production. (H) SOD activity. (I) MDA content. (J) Calcium deposition by Alizarin Red S staining (K) Intracellular calcium content. (L) ALP activity measured by ALP assay kit. * $p < 0.05$, ** $p < 0.01$, and *** $p < 0.001$ vs. the control group; # $p < 0.05$ and ## $p < 0.01$ vs. β-GP+HG group
